# Correlation between Lactate Dehydrogenase to Albumin Ratio and the Prognosis of Patients with Cardiac Arrest

**DOI:** 10.31083/j.rcm2502065

**Published:** 2024-02-18

**Authors:** Lili Ye, Jianhong Lu, Meng Yuan, Jie Min, Lei Zhong, Junfei Xu

**Affiliations:** ^1^Department of Intensive Care Unit, Huzhou Central Hospital, Affiliated Huzhou Hospital of Zhejiang University School of Medicine, 313000 Huzhou, Zhejiang, China

**Keywords:** lactate dehydrogenase to albumin ratio, cardiac arrest, clinical research, prognosis, MIMIC-IV

## Abstract

**Background:**

Cardiac arrest (CA) is a common event in the intensive care unit (ICU), which seriously threatens the prognosis of patients. Therefore, it is crucial to determine a simple and effective clinical indicator to judge the prognosis of patients after a CA for later treatments. The purpose of this study was to investigate the relationship between the lactate dehydrogenase to albumin ratio (LAR) and the prognosis of patients after a CA.

**Methods:**

The clinical data of participants was obtained from the Medical Information Mart for Intensive Care IV (MIMIC-IV, v2.0; 2008 to 2019). According to the 30-day prognosis, patients were divided into a survivors group (n = 216) and a non-survivors group (n = 304). The optimal LAR threshold was determined using restricted cubic spline (RCS), which divided patients into a high LAR group (≥15.50, n = 257) and a low LAR group (<15.50, n = 263). The ICU hospitalization and 30-day accumulative survival curves of the two groups were plotted following the Kaplan–Meier survival analysis. Multivariate Cox regression was used to analyze the relationship between the LAR and the prognosis of CA patients. Receiver operating characteristic (ROC) curves were drawn to evaluate the predictive efficacy of the LAR on 30-day all-cause mortality, and sensitivity analysis was used to check the reliability of the findings.

**Results:**

A total of 520 patients with CA were enrolled and the 30-day mortality was 58.46%. The LAR in the non-survivors group was higher than in the survivors group. The RCS showed a linear trend relationship between the LAR and the mortality risk in patients during their ICU stay and 30 days; moreover, as the LAR increased, so did the risk of mortality. The Kaplan–Meier survival curve showed that compared with the low LAR group, the cumulative survival rates of ICU hospitalization and 30 days were lower in the high LAR group among CA patients (*p* < 0.001). Multivariate Cox regression analysis showed that an elevated LAR (≥15.50) was an independent risk factor for mortality during ICU stay and 30 days (*p* < 0.005). ROC analysis suggested that the LAR was superior to the sequential organ failure assessment (SOFA) score in predicting the 30-day all-cause mortality in CA patients (area under the curve (AUC) = 0.676, 95% confidence interval [CI]: 0.629–0.723). To verify the reliability of our findings, we performed sensitivity analyses and found that the findings were reliable.

**Conclusions:**

An elevated LAR might be a predictor of mortality in patients following a CA during ICU hospitalization and 30 days, thereby it can be used to provide a reference for the clinical management of these patients.

## 1. Introduction

Cardiac arrest (CA), defined as the sudden cessation of the myocardium, leads to the interruption of blood circulation in the whole body, and then, progresses to sudden cardiac death. CA is a primary public health problem, with high levels of morbidity and mortality worldwide [1, 2]. In recent years, the management and treatment of post-CA patients has made progress, yet the overall prognosis is still poor due to the onset of pathological changes, such as severe systemic ischemia hypoxia–reperfusion injury [3], systemic inflammation response, and multiple organ dysfunction [4]. In the intensive care unit (ICU), about 0.5 percent to 5 percent of critically ill patients will experience a CA, and although approximately 50 percent will recover spontaneous circulation, only 15 percent survive until hospital discharge [5]. Therefore, it is of great significance to further strengthen and carry out research concerning CA.

Lactate dehydrogenase (LDH), a marker of tissue and organ hypoperfusion, is a key enzyme in glycolysis, by catalyzing the transformation of pyruvate to lactate. Elevated LDH levels in critically ill patients have been reported in the literature to indicate a poor outcome [6, 7]. Albumin is synthesized by the liver and has important physiological functions, such as anti-inflammatory and antioxidant activities, scavenging free radicals, and maintaining plasma osmolality. When the body is infected or hit, the consumption of albumin increases, and studies have shown that lower albumin levels are associated with increased mortality in patients with various diseases [8, 9, 10]. In addition, multiple studies have shown that elevated LDH and decreased albumin levels are associated with poor outcomes in CA patients [10, 11, 12]. The lactate dehydrogenase to albumin ratio (LAR) is a novel disease prognostic marker, which reflects the balance between LDH and albumin. Previous research has revealed that the LAR provides a high predictive effect for disease severity and poor prognosis in tumors [13], pneumonia [14], sepsis [15], and other diseases.

However, to date, no reports have linked the LAR to the prognosis of CA patients. Therefore, we investigated whether there was an association between the LAR levels at ICU admission and mortality in CA patients, to determine a simple and effective prognostic indicator to guide the clinical management of these patients.

## 2. Methods

### 2.1 Data Source

Data were obtained from a database, Medical Information Mart for Intensive Care IV (MIMIC-IV, version 2.0) database, established by the Beth Israel Deaconess Medical Center and MIT Affiliate Review. Two authors (LZ, LLY) have been certified to use this database by completing an online training course from the National Institutes of Health (certification number: 36142713, 51832843).

This database was previously approved by the Institutional Review Board. Informed consent for the study was not required because the retrospective design lacked direct patient intervention; moreover, the patient information in the database was anonymous. This study was reported under the Strengthening the Reporting of Observational Studies in Epidemiology (STROBE) guidelines [16]. All methods were carried out in compliance with the 2002 Helsinki Declaration.

### 2.2 Population

All research subjects were recruited from the MIMIC-IV database and diagnosed with CA. The inclusion criteria were: (1) CA patients extracted using structured query language (SQL) queries incorporating International Classification of Diseases (ICD) (including ICD code “4275%” for ICD-9 and ICD codes “I46%”, “I9712”, “I97120”, “I97121”, “I9771”, “I97710”, and “I97711” for ICD-10). (2) Age ≥18 years old. (3) First ICU admission. The exclusion criterion was patients with missing vital information (e.g., LDH, albumin, etc.).

### 2.3 Research Methods and Outcomes

Data included demographic, laboratory parameters, comorbidities, and treatments of each patient in the ICU (see Table 1 for details). The LAR ratio was formulated from LDH (IU/L)/albumin (g/L). Laboratory data were extracted from the first data collected within 24 h after each patient was admitted to the ICU. Treatments (e.g., defibrillation, mechanical ventilation, etc.) were derived from the data during the ICU stay.

**Table 1.** **Comparison of general information on participants**.

Variables		Overall population (n = 520)	Survivors (n = 216)	Non-survivors (n = 304)	*t/Z/χ^2^* value	*p* value
Age (years)		63.69 ± 17.58	62.30 ± 17.53	64.67 ± 17.58	–1.516	0.130
Female (n (%))		196 (37.69)	78 (36.11)	118 (38.82)	0.393	0.531
SOFA (score)		10.02 ± 4.51	8.89 ± 4.54	10.82 ± 4.33	–4.889	0.000
Charlson comorbidity index		5.84 ± 3.28	5.74 ± 3.20	5.91 ± 3.34	–0.588	0.557
LDH (IU/L)		461.50 (290.50, 869.00)	360.50 (258.00, 609.00)	534.50 (337.00, 1094.50)	–5.750	0.000
Albumin (g/L)		31.52 ± 7.44	33.66 ± 7.04	30.00 ± 7.34	5.704	0.000
LAR		15.25 (8.95, 27.58)	10.81 (7.34, 20.54)	18.34 (11.10, 38.34)	–6.841	0.000
Lactic acid (mmol/L)		3.70 (2.30, 6.30)	3.45 (1.95, 4.40)	4.00 (2.70, 7.90)	–5.408	0.000
WBC (×${\mathrm{10}}^{9}$/L)		12.70 (8.50, 18.50)	12.00 (8.60, 16.55)	13.30 (8.50, 19.95)	–1.570	0.117
HB (g/L)		116.60 ± 28.19	119.06 ± 29.73	114.85 ± 26.96	1.685	0.093
PLT (×${\mathrm{10}}^{9}$/L)		199.00 (146.50, 271.00)	206.50 (156.50, 269.50)	195.00 (133.00, 273.00)	0.919	0.358
RDW (%)		15.14 ± 2.50	14.81 ± 2.56	15.38 ± 2.44	–2.599	0.010
${\mathrm{PO}}_{2}$ (mmHg)		88.00 (58.50, 169.50)	88.00 (57.50, 173.50)	88.00 (62.00, 163.00)	0.119	0.905
ALT (U/L)		69.00 (28.00, 193.50)	54.00 (25.00, 135.00)	91.50 (31.50, 239.50)	–3.257	0.001
AST (U/L)		103.00 (44.00, 310.00)	79.50 (38.50, 181.00)	147.50 (53.00, 404.00)	–4.307	0.000
TBI (mg/dL)		0.60 (0.40, 1.10)	0.60 (0.40, 0.90)	0.70 (0.40, 1.30)	–1.808	0.071
BUN (mg/dL)		8.54 (5.70, 14.60)	7.83 (5.34, 12.10)	9.26 (5.70, 16.91)	–2.465	0.014
CRE (mmol/L)		123.76 (88.40, 185.64)	106.08 (79.56, 181.22)	132.60 (88.40, 194.48)	–2.299	0.022
PT (s)		14.30 (12.40, 19.00)	13.20 (11.80, 16.40)	15.25 (12.90, 22.00)	–5.850	0.000
Anion gap (mmol/L)		19.34 ± 6.12	17.90 ± 5.39	20.37 ± 6.40	–4.636	0.000
Glucose (mmol/L)		9.33 (6.81, 13.64)	9.06 (6.72, 13.03)	9.44 (6.89, 13.92)	–0.908	0.364
Chloride (mmol/L)		102.90 ± 7.40	102.53 ± 7.02	103.16 ± 7.67	–0.961	0.337
Sodium (mmol/L)		138.28 ± 6.30	137.96 ± 5.61	138.51 ± 6.74	–0.990	0.323
Potassium (mmol/L)		4.51 ± 1.12	4.50 ± 1.11	4.52 ± 1.12	–0.141	0.888
VF (n (%))		97 (18.65)	48 (22.22)	49 (16.12)	3.100	0.078
MV (n (%))		467 (89.81)	190 (87.96)	277 (91.12)	1.374	0.241
CRRT (n (%))		62 (11.92)	20 (9.26)	42 (13.82)	2.497	0.114
IABP (n (%))		27 (5.19)	16 (7.41)	11 (3.62)	3.683	0.055
Defibrillation (n (%))		20 (3.85)	10 (4.63)	10 (3.29)	0.613	0.434
Norepinephrine (n (%))		354 (68.08)	123 (56.94)	231 (75.99)	21.070	0.000
Dobutamine (n (%))		38 (7.31)	13 (6.02)	25 (8.22)	0.907	0.341
Echocardiography (n (%))		210 (40.38)	97 (44.91)	113 (37.17)	3.139	0.076
Comorbidities (n (%))						
	Hypertension	192 (36.92)	81 (37.50)	111 (36.51)	0.053	0.818
	Diabetes	165 (31.73)	73 (33.80)	92 (30.26)	0.728	0.394
	Cerebral infarction	69 (13.27)	27 (12.50)	42 (13.82)	0.190	0.663
	Cardiogenic shock	101 (19.42)	49 (22.69)	52 (17.11)	2.512	0.113
	AMI	121 (23.27)	53 (24.54)	68 (22.37)	0.333	0.564
	AKI	432 (83.08)	174 (80.56)	258 (84.87)	1.671	0.196
	Liver cirrhosis	39 (7.50)	11 (5.09)	28 (9.21)	3.087	0.079
	Chronic kidney disease	125 (24.04)	57 (26.39)	68 (22.37)	1.118	0.290
	Malignant tumor	66 (12.69)	24 (11.11)	42 (13.82)	0.834	0.361
Length of ICU stay (days)		3.45 (1.56, 7.98)	4.88 (2.64, 9.54)	2.56 (1.03, 5.97)	6.752	0.000

SOFA, sequential organ failure assessment; LDH, lactate dehydrogenase; LAR, lactate dehydrogenase to albumin ratio; HB, hemoglobin; TBI, total bilirubin; WBC, white blood cell; PLT, platelet; CRE, creatinine; RDW, red cell distribution width; ${\mathrm{PO}}_{2}$, oxygen partial pressure; ALT, alanine transaminase; AST, aspartate aminotransferase; BUN, blood urea nitrogen; PT, prothrombin time; VF, ventricular fibrillation; MV, mechanical ventilation; CRRT, continuous renal replacement therapy; IABP, intra-aortic balloon pump; AMI, acute myocardial infarction; AKI, acute kidney injury; ICU, intensive care unit.

According to the 30-day prognosis (from admission to ICU), patients were divided into a survivors group (n = 216) and a non-survivors group (n = 304). The optimal LAR threshold was determined using the restricted cubic spline (RCS), which divided patients into the high LAR group (≥15.50, n = 257) and the low LAR group (<15.50, n = 263).

The primary outcome variables were all-cause mortality during ICU hospitalization and 30 days.

### 2.4 Statistical Analysis

Measurement data were calculated using a *t*-test/non-parametric test and expressed as ($\bar{x}\pm s$)/M ($P_{25}$, $P_{75}$). Count data were calculated using the χ^2^ test and expressed as percentages (%).

The optimal LAR threshold was determined by RCS according to the LAR and the patient’s 30-day prognosis, which were used to divide patients into high and low LAR groups. The Kaplan–Meier (K-M) survival analysis was used to plot the survival curves of the two groups during ICU hospitalization and 30 days.

Variables with a *p* < 0.10 in the univariate analysis were included in the multivariate Cox regression analysis. Multivariate Cox regression was used to analyze the relationship between the LAR and the prognosis of the two groups during ICU hospitalization and 30 days. Model 1 was used without adjustment, while model 2 was adjusted for sequential organ failure assessment (SOFA) score, lactic acid, hemoglobin (HB), erythrocyte distribution width (RDW), alanine transaminase (ALT), aspartate aminotransferase (AST), total bilirubin (TBI), urea nitrogen (BUN), creatinine (CRE), prothrombin time (PT), and anion gap. Model 3 was adjusted for SOFA score, lactic acid, HB, RDW, ALT, AST, TBI, BUN, CRE, PT, anion gap, norepinephrine, liver cirrhosis, ventricular fibrillation (VF), ventricular fibrillation (IABP), and echocardiography.

The receiver operating characteristic curve (ROC) was drawn to calculate the area under the curve (AUC) to evaluate the predictive efficacy of the LAR on 30-day all-cause mortality.

Stata14.0 (StataCorp, College Station, TX, USA) and R language (vR-4.0.3, https://www.r-project.org/) were utilized for analysis and statistical significance was defined as *p* < 0.05.

Considering that some factors may affect the stability of the results, such as albumin infusions 3 days before admission to the ICU, and malignancy may have an effect on the LAR, a reduced synthetic ability of liver function in patients with liver cirrhosis, and increased loss of albumin in patients with chronic kidney disease may also have an impact on the LAR. After excluding these participants, we performed a sensitivity analysis to check their reliability.

## 3. Results

### 3.1 Study Population

**Fig. 1.**
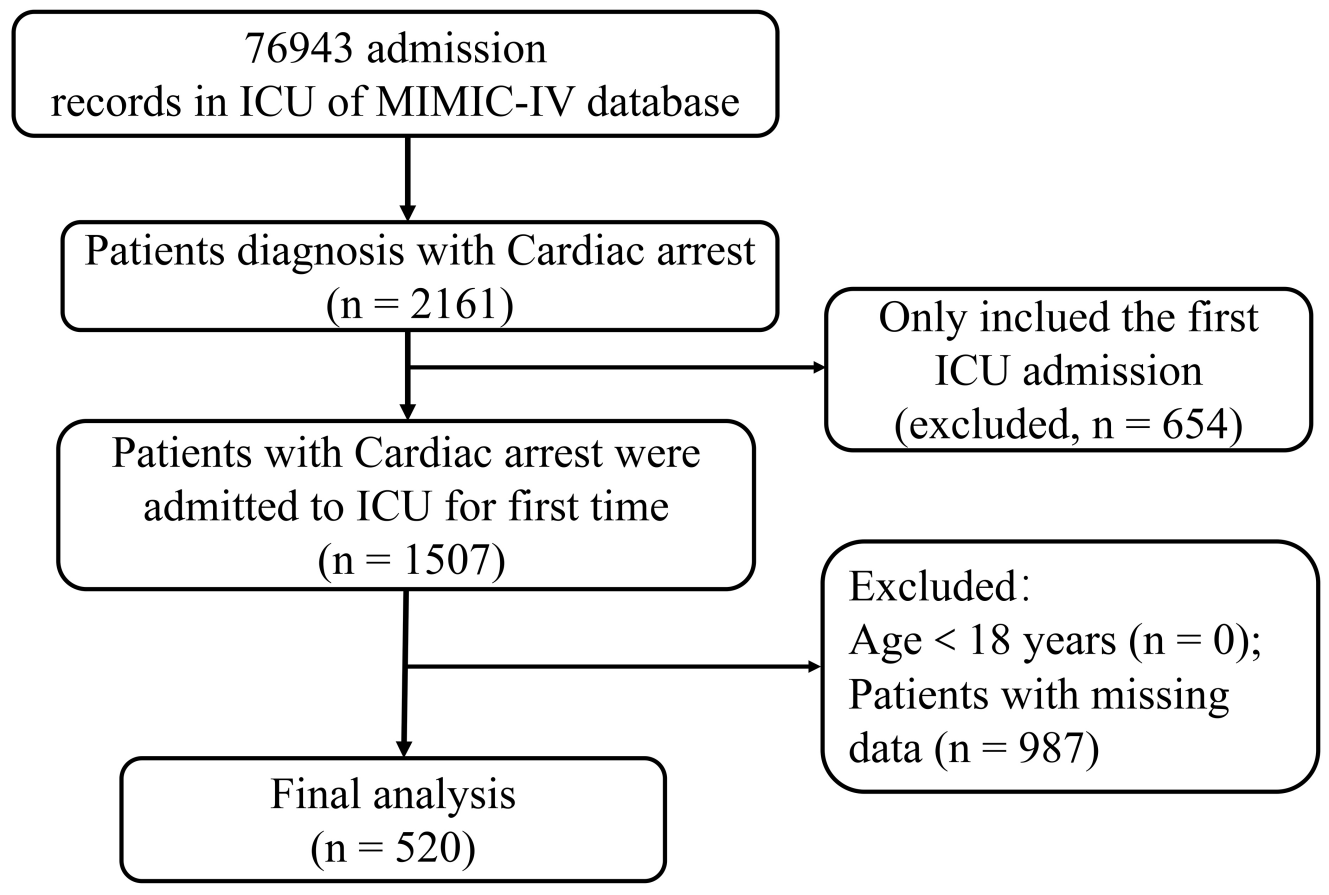
**Enrollment of research patients**. ICU, intensive care unit; MIMIC-IV, Medical Information Mart for Intensive Care IV.

A total of 520 patients who had suffered a CA were enrolled; the specific research flow chart is presented in Fig. 1. The average age was (63.69 ± 17.58) years old, and 37.69% were female, while the 30-day mortality was 58.46%. Compared with the survivors group, the SOFA score, LDH, LAR, lactic acid, RDW, ALT, AST, BUN, CRE, PT, anion gap, and norepinephrine were significantly higher in the non-survivors group. Conversely, albumin and length of ICU stay in the survivors group were significantly higher than in the non-survivors group (*p* < 0.05), as shown in Table 1.

### 3.2 RCS Analysis

**Fig. 2.**
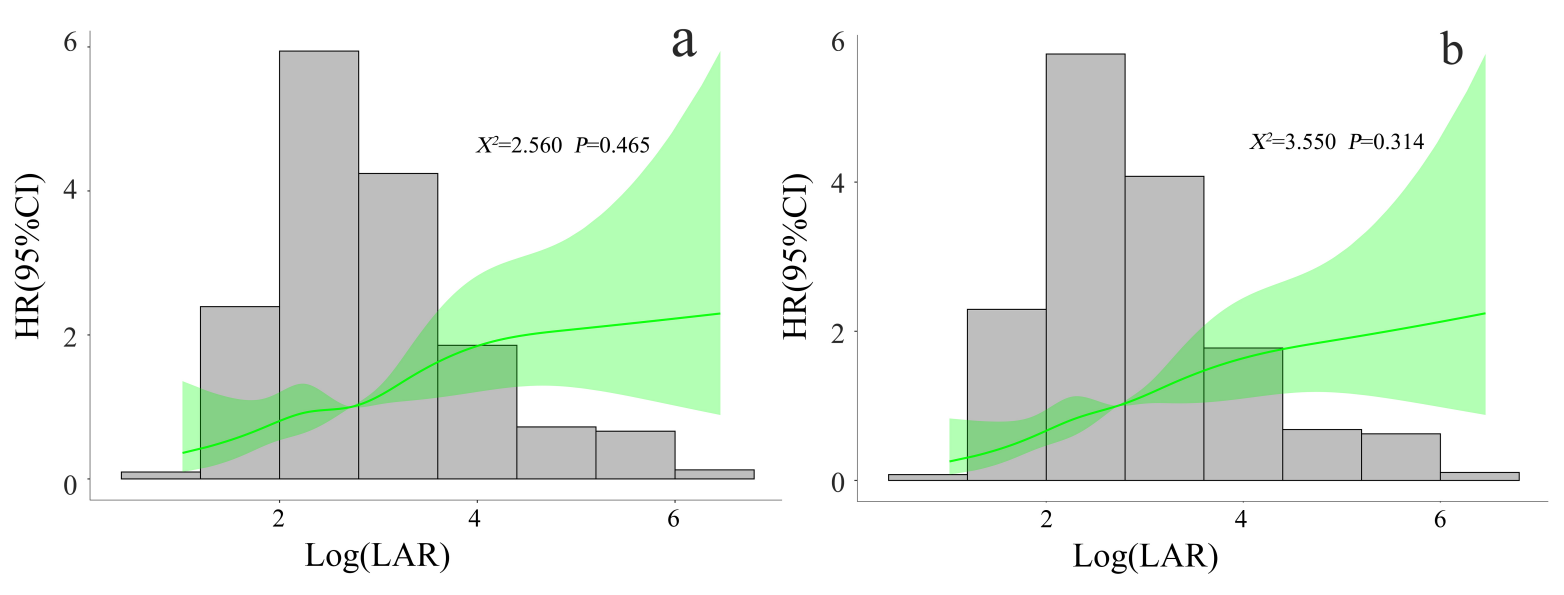
**Correlation between LAR and mortality risk in CA patients during ICU stay (a) and 30 days (b)**. RCS adjusted for SOFA score, lactic acid, HB, RDW, ALT, AST, TBI, BUN, CRE, PT, anion gap, norepinephrine, liver cirrhosis, VF, IABP, and echocardiography. LAR, lactate dehydrogenase to albumin ratio; CA, cardiac arrest; ICU, intensive care unit; RCS, restricted cubic spline; HR, hazard ratio; 95% CI, 95% confidence interval; SOFA, sequential organ failure assessment; HB, hemoglobin; RDW, red cell distribution width; ALT, alanine transaminase; AST, aspartate aminotransferase; TBI, total bilirubin; BUN, blood urea nitrogen; CRE, creatinine; PT, prothrombin time; VF, ventricular fibrillation; IABP, intra-aortic balloon pump.

RCS showed a linear trend relationship between the LAR and the mortality risk in CA patients during ICU stay and 30 days (χ^2^ = 2.560, *p* = 0.465; χ^2^ = 3.550, *p* = 0.314). When Log (LAR) was 2.741, its hazard ratio (HR) was 1. When Log (LAR) was 2.741, LAR was 15.50 (optimal threshold); therefore, as the LAR increased, so did the risk of all-cause mortality (Fig. 2).

Patients were split into high LAR (≥15.50, n = 257) and low LAR (<15.50, n = 263) groups according to the optimal threshold. The all-cause mortality during ICU hospitalization and 30 days were higher in the high LAR group than in the low LAR group (χ^2^ = 36.574, *p* = 0.000; χ^2^ = 28.047, *p* = 0.000), as shown in Table 2.

**Table 2.** **Comparing mortality rates between the two groups**.

Group	ICU hospitalization all-cause mortality				30-day all-cause mortality			
	Survivors (n = 266)	Non-survivors (n = 254)	χ ^2^	*p*	Survivors (n = 216)	Non-survivors (n = 304)	χ ^2^	*p*
Low LAR (n = 263)	169 (64.26)	94 (35.74)	36.574	0.000	139 (52.85)	124 (47.15)	28.047	0.000
High LAR (n = 257)	97 (37.74)	160 (62.26)			77 (29.96)	180 (70.04)		

LAR, lactate dehydrogenase to albumin ratio; ICU, intensive care unit.

### 3.3 K‑M Curve 

**Fig. 3.**
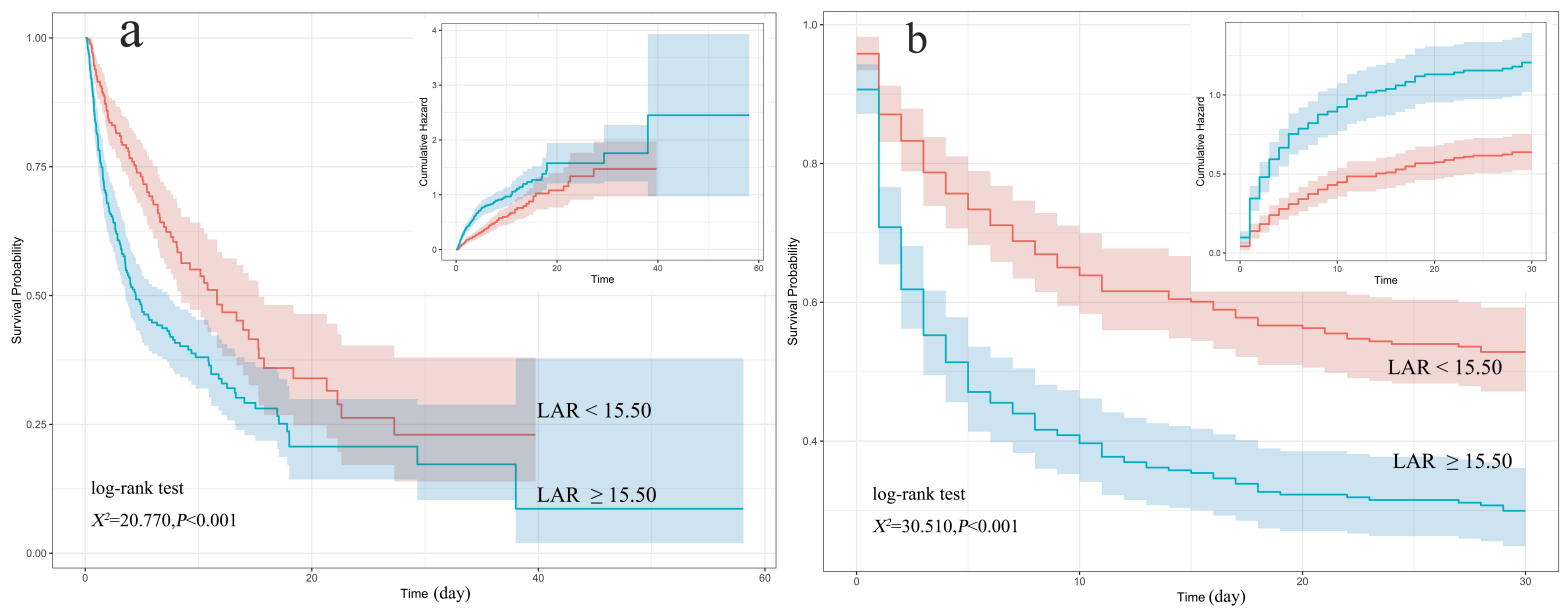
**K-M curve of patients after CA during ICU hospitalization (a) and 30 days (b)**. LAR, lactate dehydrogenase to albumin ratio; K–M, Kaplan–Meier; CA, cardiac arrest; ICU, intensive care unit.

Compared with the low LAR group, the high LAR group had lower ICU hospitalization and 30-day cumulative survival rates (log-rank test, χ^2^ = 20.770, χ^2^ = 30.510, all *p* < 0.001), as shown in Fig. 3.

### 3.4 COX Analysis

Taking the low LAR group as the baseline group, the all-cause mortality during ICU hospitalization and 30 days in the high LAR group were 1.795 (1.391–2.317) and 1.911 (1.499–2.437), respectively. After adjusting for potential confounding factors, the multivariate Cox analysis showed that an elevated LAR (≥15.50) was an independent risk factor for mortality in CA patients during ICU hospitalization and 30 days, while its HRs (95% confidence interval [CI]) were 1.530 (1.155–2.026) and 1.601 (1.220–2.101), respectively, as shown in Table 3.

**Table 3.** **Cox analysis during ICU hospitalization and 30 days**.

LAR		Model 1			Model 2			Model 3		
		HR value	95% CI	*p* value	HR value	95% CI	*p* value	HR value	95% CI	*p* value
ICU all-cause mortality										
	Low LAR	1.0			1.0			1.0		
	High LAR	1.795	1.391–2.317	0.000	1.569	1.186–2.076	0.002	1.530	1.155–2.026	0.003
30-day all-cause mortality										
	Low LAR	1.0			1.0			1.0		
	High LAR	1.911	1.499–2.437	0.000	1.626	1.241–2.132	0.000	1.601	1.220–2.101	0.001

Model 1 without adjustment. Model 2 adjusted for lactic acid, HB, RDW, ALT, AST, TBI, BUN, CRE, PT, anion gap, and SOFA score. Model 3 adjusted for, lactic acid, HB, RDW, ALT, AST, TBI, BUN, CRE, PT, anion gap, norepinephrine, liver cirrhosis, VF, IABP, echocardiography, and SOFA score. HR, hazard ratio; 95% CI, 95% confidence interval; LAR, lactate dehydrogenase to albumin ratio; ICU, intensive care unit; HB, hemoglobin; RDW, red cell distribution width; ALT, alanine transaminase; AST, aspartate aminotransferase; TBI, total bilirubin; BUN, blood urea nitrogen; CRE, creatinine; PT, prothrombin time; VF, ventricular fibrillation; IABP, intra-aortic balloon pump; SOFA, sequential organ failure assessment.

### 3.5 Predictive Efficacy of LAR on 30-Day All-Cause Mortality 

**Fig. 4.**
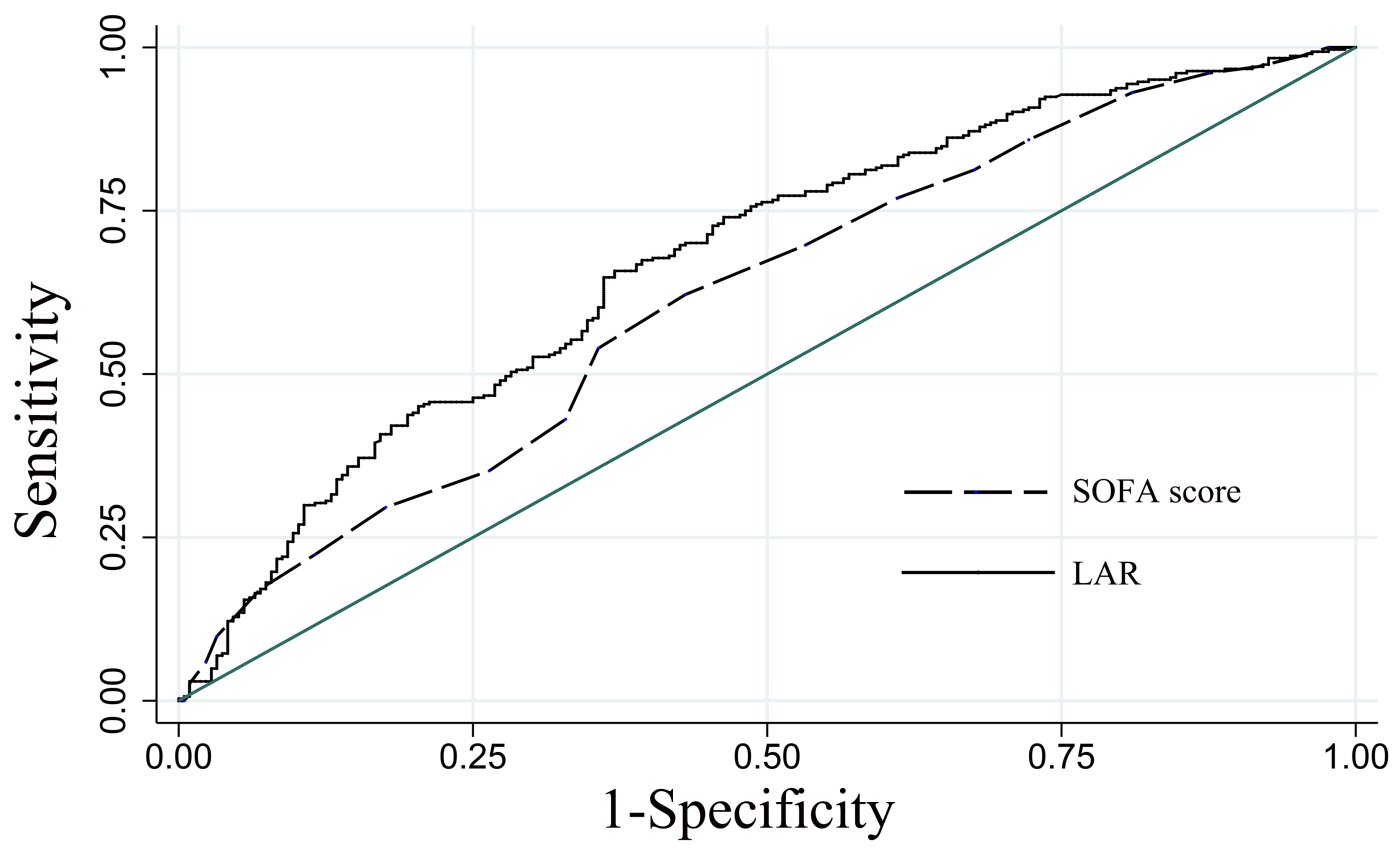
**ROC analysis of the predictive efficacy of LAR on 30-day all-cause mortality in patients with CA**. LAR, lactate dehydrogenase to albumin ratio; SOFA, sequential organ failure assessment; ROC, receiver operating characteristic curve; CA, cardiac arrest.

The ROC was used to evaluate the predictive efficacy of the LAR on 30-day all-cause mortality in patients with CA. Compared with the SOFA disease severity scoring system (AUC = 0.619, 95% CI: 0.570–0.668), the LAR (AUC = 0.676, 95% CI: 0.629–0.723) was slightly better than the SOFA score, as shown in Fig. 4.

### 3.6 Results Reliability Analysis

We performed four sensitivity analyses after excluding patients who had received an albumin infusion in the 3 days prior to ICU admission, alongside patients with malignancy, cirrhosis, and chronic kidney disease. We found that the LAR was still significantly associated with poor prognosis in CA patients (as **Supplementary Tables 1,2,3,4**).

## 4. Discussion

Based on the fact that high LDH and low albumin levels are associated with poor prognosis in CA patients, this study investigated the effects of using the LAR on the prognosis of patients after a CA. In our research, the LAR was higher in the non-survivors than in the survivors. The all-cause mortality risk during ICU hospitalization and 30 days increased accordingly as the LAR increased. The cumulative survival rates during ICU hospitalization and 30 days were lower in the high LAR group (*p* < 0.001). Furthermore, elevated LAR (≥15.50) was an independent risk factor for all-cause mortality during ICU hospitalization and 30 days (*p* < 0.005). The results may help to identify high-risk patients and assist with their clinical management.

CA is a global health problem. Thus, assessing the prognosis of patients with CA is a very complex task that requires a comprehensive judgment based on clinical, biochemical, neurophysiological, and imaging studies [17]. However, the detection of relative serological indicators is expensive, time-consuming, and difficult to promote in clinical practice. As a new disease prognostic marker, the LAR is easy to obtain, low in cost, strong in applicability, and easy to apply in clinical practice. Recently, several studies have shown that a high LAR was associated with increased mortality in many critical illnesses. A study found that the LAR was an independent prognostic indicator for patients with nasopharyngeal carcinoma, which was more predictive than using LDH or albumin and more accurate than the current staging system for nasopharyngeal carcinoma [13]. Meanwhile, Jeon found that the LAR could predict in-hospital mortality earlier in critical infection patients (odds ratio (OR) = 1.001, 95% CI: 1.000–1.002) [15]. In addition, the LAR serves as a new index to measure systemic inflammation and nutritional status, whereby a higher LAR (≥5.93) was correlated with the occurrence of cognitive dysfunction after ischemic stroke (OR = 2.003, 95% CI: 1.305–3.074) [18]. However, the LAR is rarely reported in CA patients. Therefore, we analyzed the association between the LAR and the prognosis of patients after a CA and confirmed that an elevated LAR could be used to predict short-term mortality risk.

The mechanism of the association between the LAR and poor prognosis in patients after a CA has not been fully elucidated. However, it is well known that systemic ischemia–hypoxic reperfusion injury is the link between the occurrence and development of post-CA patients [19, 20]. LDH is a cytoplasmic enzyme that is expressed in blood cells, the heart, the brain, muscle, and other body tissues. It is one of the key enzymes in the glycolysis pathway and converts pyruvate into lactic acid during hypoxic injury [21]. LDH is rapidly released into the peripheral blood after tissue cell ischemia–hypoxia injury and is a useful biomarker of cell damage [22]. Park *et al*. [6] showed that at each time point after post-cardiopulmonary resuscitation, the median LDH in the group with poor neurological prognosis was significantly higher than in the group with good neurological prognosis; thus, the inhibition of LDH was considered to have neuroprotective effects in ischemic stroke [23]. Human serum albumin has anti-inflammatory properties and protective effects in reducing ischemia–reperfusion injury [24]. Systemic ischemia–hypoxia–reperfusion injuries after CA resuscitation produce a variety of endotoxins and free radicals, while albumin can act as a scavenger for oxygen free radicals and reactive nitrogen through its binding and transport capacity to reduce organ damage [25]. In addition, albumin levels are a biochemical indicator of nutritional status, and malnutrition is considered to be one of the factors associated with a poor prognosis in seriously ill patients [26]. Several studies have shown that decreased albumin levels after cardiopulmonary resuscitation were independently associated with increased mortality [10, 12, 27].

The LAR reflects the ratio of LDH to albumin in peripheral blood, whereby an increase in the LAR is related to an increase in LDH or (and) a relative decrease in albumin, thereby indicating that the body is in an imbalanced state, which can simultaneously reflect tissue ischemia, hypoxia, and nutritional status. This may be more informative than the predictive value of LDH or albumin alone [28]. Our study showed that an elevated LAR might be linked to short-term mortality in CA patients and could be used as a supplementary prognostic factor. However, the specific mechanism needs further study.

The SOFA score has been successfully applied to assess the severity and predict the prognosis of critically ill patients. A study of 231 out-of-hospital CA patients with the return of spontaneous circulation found that an elevated SOFA score on admission was an independent predictor of 30-day all-cause mortality and poor neurological prognosis (OR = 0.68, 95% CI: 0.50–0.79; OR = 0.79, 95% CI: 0.69–0.90) [29]. This study compared the predictive efficacy of the LAR and SOFA score using the ROC curve and found that the LAR (AUC = 0.676, 95% CI: 0.629–0.723) was slightly better than the SOFA score (AUC = 0.619, 95% CI: 0.570–0.668). However, the evaluation of the SOFA score is complicated and difficult to obtain in real-time, while the LAR is simple to obtain clinically. Therefore, the LAR can be used as a predictive indicator for the prognosis of patients after a CA. The LAR, as a prognostic biomarker, can help identify high-risk patients and help clinicians make medical decisions to improve outcomes.

Based on the MIMIC database, this is a big sample study that reflects the real clinical world, while it might also be the first to evaluate the link between the LAR and the prognosis of CA patients. However, there were some limitations: First, this study was a single-center retrospective observational study, meaning several of the important prognostic indicators, such as cerebral performance category (CPC) and witnesses of CA were not available in the MIMIC database; thus, the introduction of potential bias was difficult to avoid. Second, the MIMIC-IV database does not provide data that distinguishes between in-hospital and out-of-hospital CA patients, meaning our study did not distinguish between these two populations. Furthermore, this paper only studied the LAR data at the time of admission to the ICU and did not dynamically observe the impact of the LAR levels on the mortality of patients after a CA, therefore, future studies are needed to verify our results. Finally, some comorbidities, such as cirrhosis, malignancy, and other confounding factors, may affect the results. To verify the reliability of our findings, we performed several sensitivity analyses and found that the findings were reliable. However, these findings are exploratory and multicenter prospective studies need to be designed to evaluate and confirm these data.

## 5. Conclusions

Elevated LAR (≥15.50) was a predictor of mortality in patients during ICU hospitalization and 30 days after a CA. It has strong clinical practicability and can assist clinicians in evaluating the prognosis of patients after a CA.

## Data Availability

The data that support the findings of this study are available from MIMIC-IV (https://mimic-iv.mit.edu) database but restrictions apply to the availability of these data, which were used under license for the current study, and are not publicly available. However, data are available from the MIMIC-IV dataset with permission of Massachusetts Institute of Technology (MIT) and Beth Israel Deaconess Medical Center (BIDMC).

## Abbreviations

CA, cardiac arrest; ICU, intensive care unit; LAR, lactate dehydrogenase to albumin ratio; MIMIC, Medical Information Mart for Intensive Care; RCS, restricted cubic splines; ROC, receiver operating characteristic curve; AUC, area under the curve; SOFA, sequential organ failure assessment; LDH, lactate dehydrogenase; WBC, white blood cell; HB, hemoglobin; PLT, platelet; TBI, total bilirubin; CRE, creatinine; RDW, red cell distribution width; ${\mathrm{PO}}_{2}$, oxygen partial pressure; ALT, alanine transaminase; AST, aspartate aminotransferase; BUN, blood urea nitrogen; PT, prothrombin time; MV, mechanical ventilation; CRRT, continuous renal replacement therapy; AMI, acute myocardial infarction; AKI, acute kidney injury; VF, ventricular fibrillation; IABP, intra-aortic balloon pump.

## Supplementary Material

Supplementary material associated with this article can be found, in the online version, at https://doi.org/10.31083/j.rcm2502065.
