# Supplementary material for: Correlation between Lactate Dehydrogenase to Albumin Ratio and the Prognosis of Patients with Cardiac Arrest
Source: Rev Cardiovasc Med. 2024 Feb 18;25(2):65. doi: 10.31083/j.rcm2502065 (PMC11263156; doi:10.31083/j.rcm2502065)
Supplement: Supplementary file 1 [file 2153-8174-25-2-065-s1.pdf]

Supplementary Table 1 Sensitivity analysis after removing patients who had received human serum albumin infusion 3 days before ICU admission

LAR	Model I			Model II			Model III		
	HR value	95%CI	*P* value	HR value	95%CI	*P* value	HR value	95%CI	*P* value
**ICU all-cause mortality**									
Low LAR	1.0			1.0			1.0		
High LAR	1.859	1.436~2.405	0.000	1.623	1.220~2.158	0.001	1.589	1.195~2.112	0.001
**30-day all-cause mortality**									
Low LAR	1.0			1.0			1.0		
High LAR	1.967	1.540~2.514	0.000	1.674	1.273~2.202	0.000	1.648	1.252~2.169	0.000

Model I adjusted for nothing

Model II adjusted for SOFA score, lactic acid, HB, RDW, ALT, AST, TBI, BUN, CRE, PT, anion gap.

Model III adjusted for SOFA score, lactic acid, HB, RDW, ALT, AST, TBI, BUN, CRE, PT, anion gap, norepinephrine, liver cirrhosis, VF, IABP, echocardiography.

LAR lactate dehydrogenase to albumin ratio

Supplementary Table 2 Sensitivity analysis after removing patients with malignant tumor

LAR	Model I			Model II			Model III		
	HR value	95%CI	*P* value	HR value	95%CI	*P* value	HR value	95%CI	*P* value
**ICU all-cause mortality**									
Low LAR	1.0			1.0			1.0		
High LAR	1.768	1.344~2.324	0.000	1.469	1.082~1.994	0.014	1.486	1.093~2.021	0.012
**30-day all-cause mortality**									
Low LAR	1.0			1.0			1.0		
High LAR	1.930	1.486~2.507	0.000	1.566	1.166~2.104	0.003	1.591	1.184~2.139	0.002

Model I adjusted for nothing

Model II adjusted for SOFA score, lactic acid, HB, RDW, ALT, AST, TBI, BUN, CRE, PT, anion gap.

Model III adjusted for SOFA score, lactic acid, HB, RDW, ALT, AST, TBI, BUN, CRE, PT, anion gap, norepinephrine, liver cirrhosis, VF, IABP, echocardiography.

LAR lactate dehydrogenase to albumin ratio

Supplementary Table 3 Sensitivity analysis after removing patients with cirrhosis

LAR	Model I			Model II			Model III		
	HR value	95%CI	*P* value	HR value	95%CI	*P* value	HR value	95%CI	*P* value
**ICU all-cause mortality**									
Low LAR	1.0			1.0			1.0		
High LAR	1.890	1.447~2.469	0.000	1.750	1.305~2.345	0.000	1.677	1.250~2.249	0.001
**30-day all-cause mortality**									
Low LAR	1.0			1.0			1.0		
High LAR	2.020	1.565~2.608	0.000	1.802	1.356~2.393	0.000	1.749	1.315~2.326	0.000

Model I adjusted for nothing

Model II adjusted for SOFA score, lactic acid, HB, RDW, ALT, AST, TBI, BUN, CRE, PT, anion gap.

Model III adjusted for SOFA score, lactic acid, HB, RDW, ALT, AST, TBI, BUN, CRE, PT, anion gap, norepinephrine, liver cirrhosis, VF, IABP, echocardiography.

LAR lactate dehydrogenase to albumin ratio

Supplementary Table 4 Sensitivity analysis after removing patients with chronic kidney disease

LAR	Model I			Model II			Model III		
	HR value	95%CI	*P* value	HR value	95%CI	*P* value	HR value	95%CI	*P* value
**ICU all-cause mortality**									
Low LAR	1.0			1.0			1.0		
High LAR	1.740	1.303~2.323	0.000	1.544	1.120~2.128	0.008	1.577	1.142~2.177	0.006
**30-day all-cause mortality**									
Low LAR	1.0			1.0			1.0		
High LAR	1.771	1.342~2.338	0.000	1.497	1.094~2.048	0.012	1.515	1.105~2.078	0.010

Model I adjusted for nothing

Model II adjusted for SOFA score, lactic acid, HB, RDW, ALT, AST, TBI, BUN, CRE, PT, anion gap.

Model III adjusted for SOFA score, lactic acid, HB, RDW, ALT, AST, TBI, BUN, CRE, PT, anion gap, norepinephrine, liver cirrhosis, VF, IABP, echocardiography.

LAR lactate dehydrogenase to albumin ratio
